# Key factors leading to fatal outcomes in COVID-19 patients with cardiac injury

**DOI:** 10.1038/s41598-021-82396-w

**Published:** 2021-02-18

**Authors:** Yiyu He, Xiaoxin Zheng, Xiaoyan Li, Xuejun Jiang

**Affiliations:** 1 Department of Cardiology, Renmin Hospital of Wuhan University, Wuhan, 430060 PR China; 2 Cardiovascular Research Institute, Wuhan University, Wuhan, PR China; 3 Hubei Key Laboratory of Cardiology, Wuhan, PR China

**Keywords:** Cardiology, Risk factors

## Abstract

Cardiac injury among patients with COVID-19 has been reported and is associated with a high risk of mortality, but cardiac injury may not be the leading factor related to death. The factors related to poor prognosis among COVID-19 patients with myocardial injury are still unclear. This study aimed to explore the potential key factors leading to in-hospital death among COVID-19 patients with cardiac injury. This retrospective single-center study was conducted at Renmin Hospital of Wuhan University, from January 20, 2020 to April 10, 2020, in Wuhan, China. All inpatients with confirmed COVID-19 (≥ 18 years old) and cardiac injury who had died or were discharged by April 10, 2020 were included. Demographic data and clinical and laboratory findings were collected and compared between survivors and nonsurvivors. We used univariable and multivariable logistic regression methods to explore the risk factors associated with mortality in COVID-19 patients with cardiac injury. A total of 173 COVID-19 patients with cardiac injury were included in this study, 86 were discharged and 87 died in the hospital. Multivariable regression showed increased odds of in-hospital death were associated with advanced age (odds ratio 1.12, 95% CI 1.05–1.18, per year increase; *p* < 0.001), coagulopathy (2.54, 1.26–5.12; *p* = 0·009), acute respiratory distress syndrome (16.56, 6.66–41.2; *p* < 0.001), and elevated hypersensitive troponin I (4.54, 1.79–11.48; *p* = 0.001). A high risk of in-hospital death was observed among COVID-19 patients with cardiac injury in this study. The factors related to death include advanced age, coagulopathy, acute respiratory distress syndrome and elevated levels of hypersensitive troponin I.

## Introduction

Since the coronavirus disease 2019 (COVID-19) outbreak in December 2019, caused by the severe acute respiratory syndrome coronavirus 2 (SARS-CoV-2) has spread all over the world and resulted in considerable mortality worldwide. With the increasing number of confirmed cases and information regarding the clinical features of COVID-19, acute cardiac injury caused by COVID-19 and the high risk of in-hospital death associated with cardiac injury have generated considerable concern.

Previous studies have reported that 7.2–27.8% of COVID-19 patients had myocardial injuries, and the mortality rate was markedly higher in patients with elevated troponin I levels than in patients with normal troponin I levels^1–3^. However, the reason for the high mortality associated with cardiac injury remains unclear. Myocardial injury may only partly explain the cause of death, and there may be multiple factors involved in the death of COVID-19 patients with cardiac injury. The present study aims to analyze data from a single center in Wuhan, China, and explore the potential risk factors for in-hospital death among COVID-19 patients with cardiac injury.

## Methods

### Study participants

This retrospective cohort study included all in patients with confirmed COVID-19 (≥ 18 years old) and cardiac injury who died or were discharged from Renmin Hospital of Wuhan University between January 20, 2020 and April 10, 2020. The patients in this study were diagnosed with COVID-19 according to the World Health Organization interim guidance^4^. Patients without hypersensitive troponin I were excluded. This study was approved by the National Health Commission of China and Ethics Commission of Renmin Hospital of Wuhan University (Wuhan, China). All methods were performed in accordance with the relevant guidelines and regulations. The requirement for written informed consent was waived by the Ethics Commission of Renmin Hospital of Wuhan University (Wuhan, China). The patients reported in this manuscript have not been reported in other submissions by me or anyone else.

### Data collection

We extracted the electronic medical records of patients included in this study. The demographic characteristics (age and sex), and clinical data (symptoms, comorbidities, laboratory examinations, complications, and treatments) were independently reviewed by 2 investigators.

### Laboratory procedures

To confirm COVID-19, the Viral Nucleic Acid Kit (Health) was used to extract nucleic acids from clinical samples according to the kit instructions. Throat swab samples were obtained for SARS-CoV-2 Polymerase Chain Reaction (PCR) examination. A SARS-CoV-2 detection kit (Bioperfectus) was used to detect two target genes, including open reading frame 1ab (ORF1ab) and nucleocapsid protein (N), using real-time reverse transcriptase–polymerase chain reaction. An infection was considered laboratory-confirmed if the ORF1ab and N tests both showed positive results^5^.

Routine blood examinations included complete blood count, coagulation profile, serum biochemical tests (including renal and liver function, creatine kinase, lactate dehydrogenase, albumin, total bilirubin, hypersensitive troponin I, N-terminal pro-brain natriuretic peptide, C-reactive protein, procalcitonin, CD4 count, CD8 count, interleukin-6 (IL-6), and tumor necrosis factor α). Chest radiographs or CT scans were also acquired for all inpatients. The criteria for discharge were a temperature that had returned to normal for at least 3 days, substantial improvement in both lungs in chest CT, disappearance of clinical symptoms and two negative SARS-CoV-2 RNA tests over an interval of at least 24 h^6^.

### Definitions

Acute kidney injury was identified according to the Kidney Disease: Improving Global Outcomes definition^7^. Acute cardiac injury was diagnosed if serum levels of cardiac biomarkers (e.g., high- sensitivity cardiac troponin I) were above the 99th percentile of the upper reference limit^8^. Acute respiratory distress syndrome (ARDS) was diagnosed according to the Berlin definition^9^. Acute liver injury, defined as either an alanine aminotransferase or aspartate aminotransferase level greater than three times the upper limit of normal^10^. Coagulopathy was defined as a 3-s extension of prothrombin time or a 5-s extension of activated partial thromboplastin time^6^.

### Statistical analysis

Frequency rates and percentages are used to describe categorical variables, and median and interquartile range (IQR) values are used to describe continuous variables. Continuous variables were compared using the t test and categorical variables were compared using the χ^2^ test. Univariable and multivariable logistic regression models were used to explore the risk factors for death during hospitalization. Data were analyzed using SPSS (Statistical Package for the Social Sciences) version 13.0 software (SPSS Inc). For all the statistical analyses, *p* < 0.05 was considered statistically significant.

## Result

### Patient characteristics

A total of 187 adult patients confirmed with COVID-19 and cardiac injury were hospitalized in Renmin Hospital of Wuhan University between January 20, 2020 and April 10,2020, After excluding 14 patients who were still hospitalised or did not have available key information in their medical records, we included 173 inpatients in the final analysis. Eighty-seven patients died during hospitalization, and 86 were discharged. The median age of the 173 patients was 73.0 years (IQR 64.0–80.5), ranging from 28 to 97 years, and 111 (64.2%) were male (Table 1). The most common symptoms on admission were fever (126 patients [72.8%]), followed by cough (97 patients[56.1%]), dyspnea(60 patients[34.7%], hemoptysis (39 patients [22.5%]), fatigue or myalgia (34 patients [19.6%]), chest pain (27 patients [15.6%]), diarrhea (16 patients [9.2%]), dizziness or headache (10 patients [5.8%]) , and nausea or vomiting (6 patients [3.5%]). Hypertension (72 patients [41.6%]), diabetes (29 patients [16.8%]) and coronary heart disease (29 patients [16.8%]) were the most common comorbidities, followed by cerebrovascular disease (15 patients [8.7%]), chronic obstructive pulmonary disease (9 patients [5.2%]) and chronic kidney disease (8 patients [4.6%]). Chronic liver disease (2 patients [1.2%]), cancer (2 patients [1.2%]) and chronic heart failure (1 patient [0.6%]) were rare. A total of 133 enrolled patients (76.9%) showed bilateral involvement or ground-glass opacities on chest CT scans (Table 1).

**Table 1:** Demographics and baseline characteristics of COVID-19 patients with cardiac injury.

Characteristic	No. (%)			
	Total (N = 173)	Nonsurvivors (N = 87)	Survivors (N = 86)	*p* value
Age, median (IQR), years	73 (64–80.5)	76 (65–83)	70.5 (61.75–78.25)	.010
Sex				
Female	62 (35.8%)	29 (33.3%)	33 (38.4%)	.611
Male	111 (64.2%)	58 (66.7%)	53 (61.6%)	.635
Comorbidity				
Hypertension	72 (41.6%)	41 (47.1%)	31 (36%)	.139
Coronary heart disease	29 (16.8%)	18 (20.7%)	11 (12.8%)	.164
Chronic kidney disease	8 (4.6%)	3 (3.4%)	5 (5.8%)	.459
Diabetes	29 (16.8%)	13 (14.9%)	16 (18.6%)	.519
Heart failure	1 (0.6%)	0 (0.0%)	1 (1.2%)	.313
Chronic obstructive pulmonary disease	15 (8.6%)	9 (10.3%)	6 (7.0%)	.431
Chronic liver disease	2 (1.2%)	1 (1.1%)	1 (1.2%)	.993
Cerebrovascular disease	15 (8.7%)	10 (11.5%)	5 (5.8%)	.184
Cancer	2 (1.2%)	2 (2.3%)	0 (0.0%)	.157
Signs and symptoms				
Fever (temperature ≥ 37.3℃)	126 (72.8%)	68 (78.2%)	58 (67.4%)	.113
Cough	97 (56.1%)	39 (44.8%)	58 (67.4%)	.003
Fatigue or myalgia	34 (19.6%)	21 (24.1%)	13 (15.1%)	< .001
Shortness of breath	60 (34.7%)	34 (39.1%)	26 (30.2%)	.135
Chest pain	27 (15.6%)	19 (21.8%)	8 (9.3%)	.023
Dizziness or headache	10 (5.8%)	4 (4.6%)	6 (7.0%)	.503
Nausea and vomiting	6 (3.5%)	3 (3.4%)	3 (3.5%)	.989
Hemoptysis	39 (22.5%)	18 (20.7%)	21 (24.4%)	.557
Diarrhea	16 (9.2%)	11 (12.6%)	5 (5.8%)	.121
Bilateral distribution of patchy shadows or ground-glass opacity	133 (76.9%)	67 (77.0%)	66 (76.7%)	.967

Compared with the survivors, the nonsurvivors were older (median [range] age, 76.0 [65–83] years vs 70.5 [61.75–78.25] years; *p* < 0.05), and more likely to have chest pain (19 of 87 patients [21.8%] vs 8 of 86 patients [9.3%]; *p* < 0.05), and fatigue or myalgia (21 of 87 patients [24.1%] vs 13 of 86 patients [15.1%]; *p* < 0.05). The comorbidities were not very different between survivors and nonsurvivors (Table 1).

### Laboratory findings

There were numerous differences in laboratory findings between survivors and nonsurvivors. Compared with the survivors, the nonsurvivors showed higher leukocyte, neutrophil, lactate dehydrogenase, total bilirubin, blood urea nitrogen, hypersensitive troponin I, C-reactive protein, procalcitonin, and IL-6 levels, as well as higher levels of D-dimer and lower lymphocyte, platelet count, CD8 count and CD4 counts (all *p* < 0.05; Table 2). The prothrombin time and activated partial thrombin time were longer in nonsurvivors than in survivors, with significant differences for both (all *p* < 0.05; Table 2).

**Table 2:** Laboratory findings of COVID-19 patients with cardiac injury.

Characteristic	Median (IQR)			
	Total (N = 173)	Nonsurvivors (N = 87)	Survivors (N = 86)	*p* value
Blood routine				
Leukocyte count, × 10^9^/L	7.18 (4.83–11.09)	8.88 (5.11–12.79)	6.68 (4.70–8.83)	.005
Neutrophil count, × 10^9^/L	5.72 (3.73–9.52)	7.41 (4.18–11.49)	4.97 (3.30–6.95)	.001
Lymphocyte count, × 10^9^/L	0.7 (0.45–0.98)	0.54 (0.36–0.83)	0.76 (0.62–1.11)	.001
Monocyte count, × 10^9^/L	0.40 (0.26–0.55)	0.40 (0.25–0.53)	0.40 (0.28–0.61)	.512
Platelet count, × 10^9^/L	172 (118–217)	151 (93–206)	192.50 (146.50–227.25)	.001
Hemoglobin, g/L	125 (109–137)	129 (114–138)	123.50 (105.75–136.25)	.270
Coagulation function				
Prothrombin time, s	12.70 (11.90–13.60)	12.90 (12.07–14.30)	12.50 (11.80–13.02)	.004
Activated partial thrombin time, s	29.1 (26.30–32.77)	29.60 (27.85–33.82)	28.50 (25.80–31.70)	.006
D-dimer, mg/L	2.86 (0.90–15.71)	4.33 (1.22–19.15)	1.72 (0.82–11.70)	.003
Blood biochemistry				
Albumin, g/L	33.40 (31.60–37.00)	33.40 (31.55–36.05)	33.40 (31.57–37.22)	.388
Creatine kinase, U/L	90.00 (51.00–241.00)	104 (57.00–379.50)	80.50 (47.75–161.25)	.355
Lactate dehydrogenase, U/L	428.5 (287.25–589.00)	499 (372.25–726.25)	362 (260.50–491.75)	< .001
Alanine aminotransferase,U/L	27.00 (18.00–45.00)	26.00 (19.0–40.50)	29.00 (17.50–51.25)	.286
Aspartate aminotransferase,U/L	41.00 (27.00–59.00)	47.00 (28.00–70.00)	36.50 (23.75–52.50)	.961
Total bilirubin, μmol/L	12.80 (8.70–18.30)	14.10 (9.10–22.15)	11.15 (8.60–15.72)	.009
Blood urea nitrogen, mmol/L	7.70 (5.10–13.90)	9.36 (5.81–16.50)	6.75 (4.54–9.37)	.002
Serum creatinine, μmol/L	69.00 (55.00–112.00)	76.00 (54.50–127.00)	67.00 (54.25–94.50)	.627
Hypersensitive troponin I, ng/mL	0.15 (0.69–0.75)	0.69 (0.15–2.60)	0.079 (0.056–0.15)	< .001
N-Terminal pro-brain natriuretic peptide (NT-proBNP), pg/ml	855.50 (298.50–2560.00)	1144 (475.55–3119.50)	615.90 (217.95–2027.25)	1.000
C-reactive protein, mg/L	71.40 (37.80–148.50)	101.00 (52.72–187.42)	59.10 (30.60–108.60)	< .001
Procalcitonin, ng/mL	0.174 (0.083–0.637)	0.405 (0.119–1.365)	0.103 (0.063–0.251)	.007
CD4 count, μl^−1^	205 (128–353)	164 (104.5–280.5)	290 (174–422)	< .001
CD8 count, μl^−1^	114 (59–212)	75.50 (51.25–142.75)	156.00 (83.00–233.0)	.002
CD4/CD8	1.82 (1.17–2.91)	1.77 (1.11–2.86)	1.99 (1.20–3.99)	.844
IL-6, pg/ml	41.39 (12.25–147.63)	95.85 (40.42–605.96)	15.81 (6.35–36.00)	< .001
Tumor necrosis factors(TNFα), pg/ml	3.405 (2.91–4.76)	3.68 (2.82–4.88)	3.35 (2.92–4.76)	.383

SI conversion factors: to convert alanine aminotransferase or aspartate aminotransferase to μkat/L, multiply by 0.0167.

IQR, interquartile range; IL-6, interleukin-6.

### Complications and treatments

Common complications among the 173 patients included heart failure (130 patients [75.1%]), ARDS (91 patients [52.6%]), coagulopathy (66 patients [38.2%]), acute kidney injury (65 patients [37.6%]), and acute liver injury (31 patients [17.9%]). Nonsurvivors were more likely to have coagulopathy (45 patients [51.7%] vs 21 patients [24.4%]), ARDS (68 patients [78.1%] vs 23 patients [26.7%]), acute kidney injury (42 patients [48.3%] vs 23 [26.7%]), and acute liver injury (21 patients [24.1%] vs 10 [11.6%]) than survivors. (all *p* < 0.05; Table 3).

**Table 3:** Complications and treatments of COVID-19 patients with cardiac injury.

Characteristic	No. (%)			
	Total (N = 173)	Nonsurvivors (N = 87)	Survivors (N = 86)	*p* value
Complications				
Acute liver injury	31 (17.9%)	21 (24.1%)	10 (11.6%)	.032
Acute kidney injury	65 (37.6%)	42 (48.3%)	23 (26.7%)	.003
Heart failure	130 (75.1%)	68 (78.2%)	62 (72.1%)	.356
Coagulopathy	66 (38.2%)	45 (51.7%)	21 (24.4%)	< .001
ARDS	91 (52.6%)	68 (78.1%)	23 (26.7%)	< .001
Treatments				
Antiviral therapy	147 (85.0%)	71 (81.6%)	76 (88.4%)	.213
Antibiotic therapy	148 (85.5%)	79 (90.8%)	69 (80.2%)	.048
Corticosteroid	117 (67.6%)	66 (75.9%)	51 (59.3%)	.020
Immunoglobulin	130 (75.1%)	71 (81.6%)	59 (68.6%)	.048
Vasoconstrictive agents	75 (43.4%)	58 (66.7%)	17 (19.8%)	< .001
Continuous renal replacement therapy	31 (17.9%)	20 (23.0%)	11 (12.8%)	.080
Oxygen support				
Nasal cannula	137 (79.2%)	80 (92.0%)	57 (66.3%)	< .001
Noninvasive ventilation or high-flow nasal cannula	85 (49.1%)	56 (64.4%)	29 (33.7%)	< .001
Invasive mechanical ventilation or ECMO	28 (16.2%)	16 (18.4%)	12 (14.0%)	.428

ARDS, acute respiratory distress syndrome; ECMO, extracorporeal membrane oxygenation.

The usage rates of nasal cannula, noninvasive ventilation or high-flow nasal cannula, and invasive mechanical ventilation or ECMO were 79.2% (137 patients), 49.1% (85 patients), and 16.2% (28 patients), respectively. Compared with the survivors, the nonsurvivors required more noninvasive ventilation or high-flow nasal cannula (56 [64.4%] vs 29 [33.7%]; *p* < 0.001) (Table 3).

Antibiotic and antiviral therapy were the most commonly used treatments (148 [85.5%], and 147 [85%], respectively), followed by intravenous immunoglobulin therapy (130 [75.1%]), corticosteroids (117 [67.6%]), vasoconstrictive agents (75 [43.4%]) and continuous renal replacement therapy (31 [17.9%]). The use of corticosteroids (66 [75.9%] vs 51 [59.3%]), and vasoconstrictive agents (58 [66.7%] vs 17 [19.8%]) was more common in nonsurvivors than in survivors (all *p* < 0.05; Table 3).

### Risk factors for mortality

In the univariable analysis, the odds of in-hospital death were higher in patients with acute liver injury, acute kidney injury, ARDS, or coagulopathy. Lymphopenia, elevated leucocytes, elevated neutrophil count, elevated hypersensitive troponin I, elevated procalcitonin and a prolonged prothrombin time were also associated with death. We included 173 patients with complete data for all variables (87 nonsurvivors and 86 survivors) in the multivariable logistic regression model, and we found that advanced age, ARDS, coagulopathy, and elevated level of hypersensitive troponin I were associated with increased odds of death (Table 4). Age was associated with a 1.12-fold higher risk of death (OR: 1.12; 95% CI: 1.05–1.18), ARDS was associated with a 16.56-fold higher risk of death (OR: 16.56; 95% CI: 6.66–41.2), coagulopathy was associated with a 2.54-fold higher risk of death (OR: 2.54; 95% CI: 1.26–5.12), and hypersensitive troponin I was associated with a 4.54-fold higher risk of death (OR: 4.54; 95% CI: 1.79–11.48). (Table 4).

**Table 4:** Risk factors associated with in-hospital death.

	Univariable OR (95% CI)	*p* value	Multvariable OR (95% CI)	*p* value
**Demographics and complications of COVID-19 patients with cardiac injury**				
Age, years	1.02 (1.00–1.05)	.012	1.12 (1.05–1.18)	< .001
Acute liver injury	2.41 (1.06–5.50)	.035	1.44 (0.58–3.53)	.423
Acute kidney injury	2.55 (1.35–4.83)	.004	1.79 (0.89–3.57)	.098
Coagulopathy	3.31 (1.73–6.33)	< .001	2.54 (1.26–5.12)	.009
ARDS	13.43 (6.00–30.08)	< .001	16.56 (6.66–41.20)	< .001
**Laboratory findings of COVID-19 patients with cardiac injury**				
Leukocyte count, × 109/L	1.10 (1.02–1.18)	.007	–	–
Neutrophil count, × 109/L	1.13 (1.05–1.23)	.001	–	–
Lymphocyte count, × 109/L	0.02 (0.08–0.46)	< .001	–	–
Platelet count, × 109/L	0.99 (0.98–0.99)	.002	–	–
Prothrombin time, s	1.41 (1.11–1.79)	.005	–	–
Activated partial thrombin time, s	1.08 (1.01–1.15)	.012	–	–
D-dimer, mg/L	1.02 (1.00–1.03)	.008	–	–
Lactate dehydrogenase, U/L	1.00 (1.00–1.00)	.362	1.00 (1.00–1.00)	.01
Total bilirubin, μmol/L	1.03 (1.00–1.07)	.019	–	–
Blood urea nitrogen, mmol/L	1.06 (1.01–1.10)	.004	–	–
Hypersensitive troponin I, ng/mL	7.95 (2.64–23.87)	< .001	4.54 (1.79–11.48)	.001
C-reactive protein, mg/L	1.01 (1.00–1.01)	< .001	–	–
Procalcitonin, ng/mL	2.90 (1.55–5.42)	.001	–	–
CD8 count, μL^−1^	0.99 (0.99–0.99)	.005	–	–
CD4 count, μL^−1^	0.99 (0.99–0.99)	< .001	0.99 (0.99–1.00)	.057
IL-6, pg/mL	1.01 (1.00–1.02)	< .001	1.01 (1.00–1.02)	.017

OR, odds ratio; ARDS, acute respiratory distress syndrome.

## Discussion

In this study, we included 173 COVID-19 patients with cardiac injury and 87 of whom died. Notably, COVID-19-induced cardiac injury was associated with a high risk of death. By analyzing the risk factors for death, we found that older age, coagulopathy, ARDS and higher hypersensitive troponin I levels were associated with higher odds of in-hospital death.

According to recent studies in China, myocardial injury is independently associated with an increased risk of mortality among COVID-19 patients^1,2^. However, the reasons for the high mortality associated with cardiac injury are not well described. Advanced age has been reported as an important independent predictor of mortality in patients with COVID-19^6^. Our study confirmed that advanced age was associated with death in COVID-19 patients with cardiac injury. However, there was no difference in comorbidities between survivors and nonsurvivors. These data differ from a recent report that showed that comorbidities may be a risk factor for poor outcome^11^. Symptoms including fatigue, myalgia and chest pain were more common in patients who died. Nonsurvivors required more noninvasive ventilation or high-flow nasal cannula therapy. Major complications including ARDS, coagulopathy, acute liver injury, and acute kidney injury, occurred more often in nonsurvivors. Additionally, lymphopenia, elevated leukocyte count, elevated neutrophil count, and elevated hypersensitive troponin I were also associated with death.

In our study, nonsurvivors had higher levels of D-dimer, and a longer prothrombin time and activated partial thrombin time, but lower platelet counts than survivors. Coagulation disorders are relatively frequently encountered among COVID-19 patients, especially among those with adverse outcomes. The pathogenetic mechanisms may include endothelial dysfunction, increased consumption of platelets and the decreased production of platelets, von Willebrand factor elevation, Toll-like receptor activation, and tissue-factor pathway activation^12^. COVID-19 patients with coagulation disorders are at risk of developing disseminated intravascular coagulation (DIC), deep vein thrombosis and pulmonary embolism, and multiorgan infarcts, which increase the risk of death^13^. These observations were also reported in a previous autopsy study on SARS-CoV-1-infected patients^14^. Coagulation activation could be related to a sustained systemic pro-inflammatory cytokine release elicited by viral infections. Inflammation not only leads to the activation of coagulation, but coagulation also affects inflammatory activity, suggesting the extensive cross-talk between these two systems^15^. In a coagulation cascade, IL-6 not only increases the production of fibrinogen in the liver, but also activates the coagulation system, and infusion of a monoclonal anti–IL-6 antibody resulted in the complete abrogation of coagulation activation^16^.

In our study, the level of IL-6 was much higher in nonsurvivors than in survivors, and IL-6 level appears to be a prognostic indicator of outcome. We also found that markers of inflammatory response, such as leukocytes, neutrophil, C-reactive protein and procalcitonin were significantly increased among nonsurvivors. These abnormalities suggest that the severe inflammatory response may be associated with death among COVID-19 patients with cardiac injury. Previous studies showed that the levels of serum pro-inflammatory cytokines (IL-6 and IFN-α) and chemokines (IL-8, CXCL- 10, and CCL5) were much higher in patients with severe MERS than in patients with mild and moderate disease^17,18^. Increased levels of proinflammatory cytokines (IFN-γ, IL-1, IL-6, IL-12, and TGFβ) and chemokines (CCL2, CXCL10, CXCL9, and IL-8) were observed in severe SARS patients compared to mild individuals^19,20^. Inflammatory cytokines can be released into the circulation and induce lung epithelial and endothelial cell apoptosis, which results in vascular leakage, alveolar edema, epithelial cell proliferation and impaired tissue remodeling ultimately resulting in ARDS^21^. Inflammatory cytokines can also induce hypotension, tissue hypoxia, myocardial dysfunction, and eventually lead to multiple organ dysfunction and DIC^22^.

We also observed that the CD4 T cell and CD8 T cell counts decreased more in nonsurvivors than in survivors. These abnormalities suggest that mortality may be associated with cellular immune deficiency. Both CD4 and CD8 T cells play a critical role in clearing viruses by eliminating virus-infected cells. Reduction in CD4 and CD8 T cells were associated with severe pneumonia. A previous study showed that a dramatic loss of CD4 T cells and CD8 T cells strongly correlated with the severity of the acute phase of SARS disease in humans^23,24^. A recent pathological study also found that the peripheral CD4 and CD8 T cell counts were substantially reduced in COVID-19 patients, while their status was overactivated, which accounts for the severe immune injury, in nonsurvivors^25^. In SARS-CoV-infected mouse models, the depletion of CD4 T and CD8 T cells reduced neutralizing antibody titers in the lungs, delayed virus clearance and further enhanced immune-mediated interstitial pneumonitis^26^.

Patients with coagulation activation, cellular immune deficiency and high levels of inflammatory cytokines are more likely to experience organ injury and a higher risk of death after COVID-19 infection. In this study, the level of hypersensitive troponin I, and incidence of ARDS, acute liver injury and acute kidney injury were much higher in nonsurvivors. Further multivariable logistic regression analysis showed that advanced age, elevated hypersensitive troponin I, coagulopathy and ARDS were independently associated with an increased risk of death in COVID-19 patients with cardiac injury. These observations suggest that the causes of death among COVID-19 patients with cardiac injury may involve multiorgan dysfunction and coagulation disorders, and myocardial injury may only partly explain the cause of death. Cytokine storms and a series of immune responses, or coagulation disorders may be the pathophysiological mechanism underlying organ injury caused by COVID-19. The respiratory system is the most commonly involved system in COVID-19, and some patients can rapidly develop ARDS. Previous reports showed that the incidence of ARDS was 15.6–31%, higher than that of other organ injuries^3,27^. The main cause of ARDS may be the injury to the alveolar epithelial cells. A recent pathological study showed evident that ARDS in the lungs was caused by SARS-CoV-2 infection. A few interstitial mononuclear inflammatory infiltrates were found in heart tissue, but no other substantial myocardial damage was observed in a patient with COVID-19, indicating that there were no obvious histological changes seen in heart tissue^25^. ARDS may be the main cause of death among COVID-19 patients, which is consistent with our research on COVID-19 patients with cardiac injury. The data in this study suggested that coagulopathy and ARDS are valuable warning models for predicting mortality in COVID-19 patients with cardiac injury.

This study provides novel and valuable warning information for physicians to predict the severity of the COVID19 patients with cardiac injury, and makes it possible to identify patients with a high risk of death earlier and provide timely and active management, leading to better clinical outcomes.

## Conclusions

A high risk of in-hospital death was shown among COVID-19 patients with cardiac injury in this study. The factors related to death include advanced age, coagulopathy, acute respiratory distress syndrome and elevated levels of hypersensitive troponin I.

## Data Availability

The datasets generated during or analyzed during the current study are available from the corresponding author on reasonable request.
